# Supplementary material for: Investigation by DFT Methods of the Damage of Human Serum Albumin Including Amino Acid Derivative Schiff Base Zn(II) Complexes by IR-FEL Irradiation
Source: Int J Mol Sci. 2019 Jun 11;20(11):2846. doi: 10.3390/ijms20112846 (PMC6600442; doi:10.3390/ijms20112846)
Supplement: Supplementary file 1 [file ijms-20-02846-s001.pdf]

SUPPLEMENTARY MATERIALs SECTION

Table S1. Geometrical parameters, bond lengths (in Å), and angles (in degrees) calculated at the B3LYP/6-31G** level in ZnAHN and ZnVHN.

Parameters	ZnAHN		ZnVHN		
	Conf. 1	Conf. 2	Conf. 1	Conf. 2	Conf. 3
Bond lengths					
O22-Zn	1.909	1.899	1.924	1.904	1.915
O24-Zn	1.875	1.883	1.882	1.894	1.878
O27-Zn	2.058	2.053	2.074	2.063	2.071
N-Zn	1.956	1.960	1.962	1.963	1.958
C-O22	1.312	1.312	1.320	1.319	1.317
C=O23	1.219	1.219	1.220	1.220	1.221
Bond angles					
O22-Zn-O24	147.6	147.0	165.8	163.9	156.8
O22-Zn-O27	89.7	100.7	82.3	95.3	109.1
N-Zn-O22	88.0	88.1	86.6	87.2	87.3
N-Zn-O24	98.6	98.0	95.3	94.8	96.6
N-Zn-O27	134.5	134.8	133.6	134.8	132.6
O=C-O	125.3	125.2	124.9	124.8	124.6
Torsional angles					
C8-C9-O24-Zn	5.7	4.9	9.1	9.6	8.2
C9-O24-Zn-O22	-108.3	-106.4	-117.2	-117.4	-114.2
C9-O24-Zn-O27	133.6	130.1	118.6	114.8	124.0
C9-O24-Zn-N	-8.4	-7.4	-19.8	-20.9	-15.6
O22-Zn-O27-C29	-134.3	-13.7	-39.4	-4.1	149.0
C9-C8-C11-N	-20.5	-20.4	-9.5	-9.8	-16.0
O24-Zn-O22-C21	108.3	107.8	113.2	112.4	113.1

Table S2. Theoretical computed total energies (A.U.), zero-point vibrational energies (kJ mol^{-1}), rotational constants (GHz), entropies ($J\cdot mol^{-1} K^{-1}$), and dipole moments (Debyes) calculated with the B3LYP and MP2 methods in ZnAHN and ZnVHN.

Parameter	ZnAHN		ZnVHN		
	Conf. 1	Conf. 2	Conf. 1	Conf. 2	Conf. 3
Total energy + ZPE	-2715.254468	-2715.254615	-2793.824606	-2793.824448	-2793.828081
Free energy	-2715.305567	-2715.304614	-2793.879027	-2793.878265	-2793.882686
Rotational constants	0.476	0.511	0.346	0.358	0.350
	0.174	0.169	0.158	0.156	0.156
	0.134	0.134	0.122	0.121	0.121
Entropy					
Total	152.3	149.8	165.2	164.0	165.6
Translational	43.3	43.3	43.6	43.6	43.6
Rotational	34.6	34.6	35.1	35.1	35.1
Vibrational	74.3	71.9	86.5	85.3	86.9
Dipole moment	5.7675	6.163	4.717	5.333	5.319

Table S3. Calculated natural NBO atomic charges with the B3LYP method in ZnAHN and .ZnVHN.

Atom	ZnAHN		ZnVHN		
	Conf. 1	Conf. 2	Conf. 1	Conf. 2	Conf. 3
O22	-0.827	-0.821	-0.839	-0.830	-0.834
O24	-0.783	-0.785	-0.776	-0.783	-0.775
O27	-0.783	-0.783	-0.794	-0.789	-0.791
O23	-0.629	-0.629	-0.625	-0.626	-0.632
Zn	1.270	1.272	1.292	1.291	1.287
N	-0.631	-0.629	-0.643	-0.639	-0.643
C21	0.819	0.819	0.813	0.814	0.809

Table S4. Comparison of the calculated harmonic wavenumbers (ν^{cal}, cm^{-1}), absolute (A) and relative infrared intensities (A, %), reduced masses (μ), force constants (f, mDyne/Å), scaled wavenumbers (ν^{scal}, cm^{-1}), experimental wavenumbers by IR (ν^{exp}), and characterization obtained in the ZnAHN molecule at the B3LYP/6-31G(d,p) level.

Theoretical conf 1						Theoretical conf 2		Experimental	
ν^{cal}	A	A^{a}%	μ	f	$\nu^{scal, b}$	ν^{cal}	$\nu^{scal, b}$	ν^{exp}	Characterization
425	9	2	4.3	0.46	420	425	420	418 w	40%, δ(O22-Zn-O24) + 38%, 16b γ(CCC)
454	5	1	4.5	0.55	449	445	440	432 vw	36%, γ(C19-N) + 34%, δ(COO)
457	10	2	7.0	0.86	452	456	451	451 w	50%, ν(O24-Zn) + 31%, 16b γ(CCC)
515	19	3	6.3	0.99	509	460	455	493, 472 vw	43%, 16b γ(CCC) + 31% δ(C11-N-Zn)
533	5	1	3.9	0.65	526	514	508	516, 509 vw	68%, 16a γ(CCC)
548	83	15	1.6	0.28	541	534	527	535, 524 vw	71%, γ(O-H)
553	12	2	3.0	0.54	546	552	545	554, 544 vw	63%, 16a γ(CCC)
561	11	2	3.7	0.69	554	558	551	563 w	41% ν(C11-N) + 22%, ν(O22-Zn-O24) + 20%, δ(C=O)
576	32	6	4.8	0.94	569	574	567	588, 577 sh	38% ν(CNC) + 25%, ν(O22-Zn-O24) + 18%, δ(C=O)
604	35	6	6.4	1.37	596	601	593	594 w	70%, ν(O22-Zn-O24) + 17%, γ(C-C19-C)
642	18	3	4.8	1.17	633	640	631	651, 637 vw	41%, ν(N-Zn) + 38%, ν(O22-Zn-O24)
670	2	0	5.4	1.44	661	670	661	664 vw	63%, 6a δ(CCC) + 18%, δ(N-C19)
693	2	0	3.0	0.85	683	694	684	681 vw	85%, 16a γ(CCC, CH) mainly in C8-C9 and C10-H
765	28	5	1.4	0.47	754	765	754	746 m	95%, 17b γ(C-H)
768	10	2	3.9	1.35	757	769	758		65% δ_{as}(CCOO)
773	12	2	6.1	2.16	762	772	761	770 vw	48%, ν(O24-Zn) + 35%, δ(C7-C10) +12%, δ(CCC, CH) in benzene ring
782	4	1	4.4	1.58	771	782	771		63%, 16a γ(CCC) + 18% γ(C19-C21)
786	3	1	4.2	1.51	775	787	776	784 w	72%, 16a γ(CCC)
847	33	6	1.6	0.66	834	847	834		97%, 17b γ(C-H)
859	68	12	5.9	2.58	846	859	846	830 m	70%, δ(COO) + 13%, δ(N-C19-C33)
878	6	1	1.6	0.74	865	878	865	860 w	97%, 17a γ(C-H)
885	5	1	3.8	1.76	872	885	872	884 vw	76%, 12 δ(CCC)
917	18	3	3.2	1.60	903	917	903	929 w	65%, δ(N-C-C33)
950	2	0	1.3	0.71	936	950	936	943 sh	96%, 17a γ(C-H)
983	1	0	1.3	0.74	968	983	968		85%, 5 γ(C-H)
988	6	1	3.0	1.71	973	988	973	972 w	34%, 12 δ(CCC) + 29%, γ(C11-N) + 28%, γ(C11-H)
990	0	0	1.3	0.75	975	990	975		98%, 5 γ(C-H)
1002	33	6	1.9	1.14	987	1001	986	983 w	78%, γ(C11-H)
1037	123	22	6.5	4.12	1021	1021	1005		85%, ν(C29-O)

1061	11	2	1.9	1.26	1044	1061	1044	1040 vw	48%, δ(C19-H) + 34%, δ(C-H) in C33H_3
1072	7	1	2.1	1.41	1055	1072	1055	1064 vw	82%, 18b δ(CC, C-H) in benzene ring II
1103	113	20	1.2	0.87	1086	1097	1080	1104 m	46%, γ(OH) + 38%, δ(C-H) in C29H_3
1109	27	5	2.0	1.44	1092	1108	1091	1127 s	35%, 15 δ(C-H) + 28%, δ(C-H) in C33H_3 +13% δ(C19-H)
1129	2	0	1.9	1.40	1111	1129	1111		35%, δ(C-H) in C33H_3 + 27%, 15 δ(C-H) + 16% δ(C19-H)
1146	2	0	3.1	2.37	1128	1147	1129		70%, ν(C19-N) + 25%, ν(C19-C33)
1175	1	0	1.2	1.01	1156	1175	1156	1150 sh	97%, 9a δ(C-H)
1181	1	0	1.3	1.03	1162	1182	1163	1157 w	94%, δ(C-H) in C29H_3
1193	7	1	1.3	1.08	1174	1194	1175		96%, 9b δ(CC, C-H) mainly in C6-H
1215	64	12	1.9	1.68	1196	1215	1196	1187 s	83%, 9b δ(CC, C-H) + 15% δ(C11-H)
1247	2	0	2.0	1.84	1227	1247	1227	1222 vw	88%, δ(CC, C-H) in benzene ring
1275	25	5	2.2	2.07	1254	1275	1254	1247 w	55%, 3 δ(C-H) + 32%, ν(C-O)
1280	44	8	1.6	1.51	1259	1279	1258		82%, δ(C19-H)
1297	144	26	3.3	3.26	1276	1296	1275	1280 m	60%, ν(C21-O) + 20%, δ(C19-H) +15%, 3 δ(C-H)
1324	71	13	1.7	1.81	1303	1325	1304	1298 m	70%, δ(C20-H) + 25%, 19b ν(CC, CH)
1339	77	14	1.9	2.06	1317	1340	1318		70%, δ(C20-H) + 25%, 19b ν(CC, CH)
1368	31	6	1.3	1.46	1346	1360	1338	1340 m	70% δ(O-H) + 25% δ(C-H) in C29H_3
1375	108	19	3.7	4.14	1353	1375	1353	1360 m	52%, 14 ν(C=C) + 28% δ(C11-H) + 15%, ν(C9-O)
1395	31	6	3.1	3.57	1372	1395	1372		70%, 14 ν(C=C) + 20% δ(C11-H)
1408	14	3	1.3	1.55	1385	1408	1385	1391 m	80% δ_s(C-H) in C33H_3
1421	172	31	4.9	5.82	1398	1423	1400	1409 s	60%, ν(C9-O) + 30%, δ(CC, C-H) in benzene ring
1443	17	3	1.8	2.17	1419	1444	1420		60%, δ(C11-H) +25%, δ(C11=N) + 10%, δ(C-H) in benzene ring
1473	68	12	2.5	3.23	1424	1473	1424	1432 w	60%, 19b ν(C=C, C-H) + 25%, δ(C11-H)
1489	3	1	1.2	1.53	1439	1488	1438		85%, δ_s(C-H) in C29H_3
1495	73	13	2.2	2.84	1444	1496	1445	1457 s	68%, 19b ν(C=C, C-H) + 25%, δ_{as}(C-H) in C29H_3
1506	7	1	1.1	1.42	1455	1509	1458		92%, δ_{as}(C-H) in C29H_3
1508	3	1	1.1	1.42	1457	1512	1460		90%, δ_{as}(C-H) in C33H_3
1514	15	3	1.0	1.42	1462	1513	1461		88%, δ_{as}(C-H) in C33H_3
1519	4	1	1.0	1.42	1467	1517	1465		85%, δ_{as}(C-H) in C29H_3
1554	88	16	3.3	4.73	1500	1554	1500	1508 w	85%, 19a ν(C=C)+ δ(C-H) in benzene ring
1587	83	15	5.7	8.51	1531	1587	1531	1542 s	85%, 19a ν(C=C)
1654	3	1	6.1	9.77	1594	1654	1594		80%, 8b ν(C=C) in benzene rings
1670	20	4	6.0	9.85	1609	1670	1609		85%, 8b ν(C=C) in benzene rings
1680	554	100	6.6	10.91	1619	1681	1620	1622 vs	70%, ν(C11=N) + 20% ν(C-C) in ring I
1797	499	90	12.5	23.74	1729	1797	1729		90%, ν(C21=O)
3008	20	4	1.1	5.77	2871	3007	2870	2873 w	94%, ν(C19-H)
3049	17	3	1.0	5.69	2910	3049	2910	2905 vw	97%, ν_s(C-H) in C33H_3

3055	43	8	1.0	5.67	2915	3056	2916	2930 w	99%, ν_s(C-H) in $C29H_3$
3115	22	4	1.1	6.31	2972	3115	2972	2968 vw	90%, ν_{as}(C-H) in $C33H_3$
3131	15	3	1.1	6.27	2987	3131	2987	2985 w	90%, ν(C11-H)
3131	24	4	1.1	6.40	2987	3135	2991		92%, ν_{as}(C-H) in $C29H_3$
3164	4	1	1.1	6.48	3018	3164	3018		98%, ν_{as}(C-H) in $C33H_3$
3174	0	0	1.1	6.46	3027	3174	3027		99%, ν(C-H) in benzene rings
3178	14	3	1.1	6.47	3031	3177	3030		99%, ν(C-H) in benzene rings
3179	8	1	1.1	6.57	3032	3185	3038		99%, ν_{as}(C-H) in $C29H_3$
3185	10	2	1.1	6.51	3038	3186	3039		95%, 20b ν(C-H) in benzene ring II
3204	31	6	1.1	6.61	3056	3204	3056		95%, 20b ν(C-H) in benzene ring II
3211	19	3	1.1	6.65	3062	3210	3061		100%, ν(C-H) in benzene ring I
3212	13	2	1.1	6.66	3063	3212	3063		100%, ν(C-H) in benzene ring II
								3324s, 3275 s	ν(O-H)
3783	96	17	1.1	8.99	3602	3829	3645	3429 vs	100%, ν(O-H)

[a] Normalized to the highest value. [b] With the scaling equation: $\nu^{scal} = 3.3 + 0.9813\cdot\nu^{cal.}$ (for the 400–1450 cm^{-1} range) and $\nu^{scal} = 34.7 + 0.9429\cdot\nu^{cal.}$ (for the 1450–3800 cm^{-1} range)

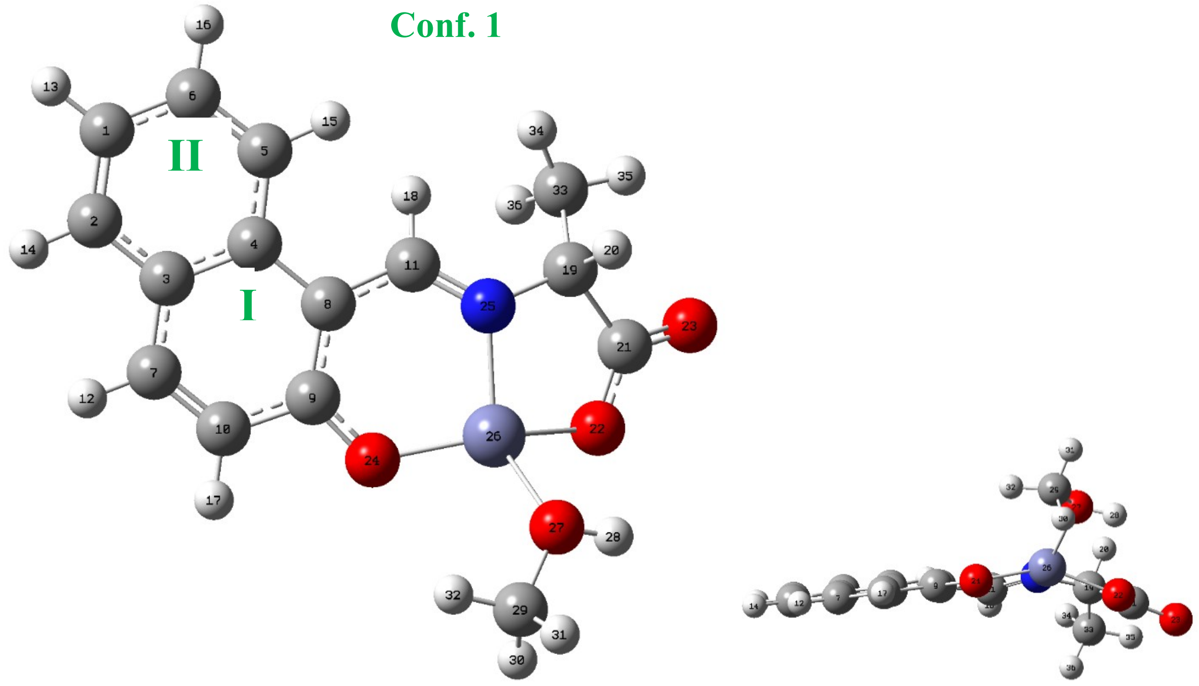

E = -2715.254468 AU (*G* = **-2715.305567 AU**)

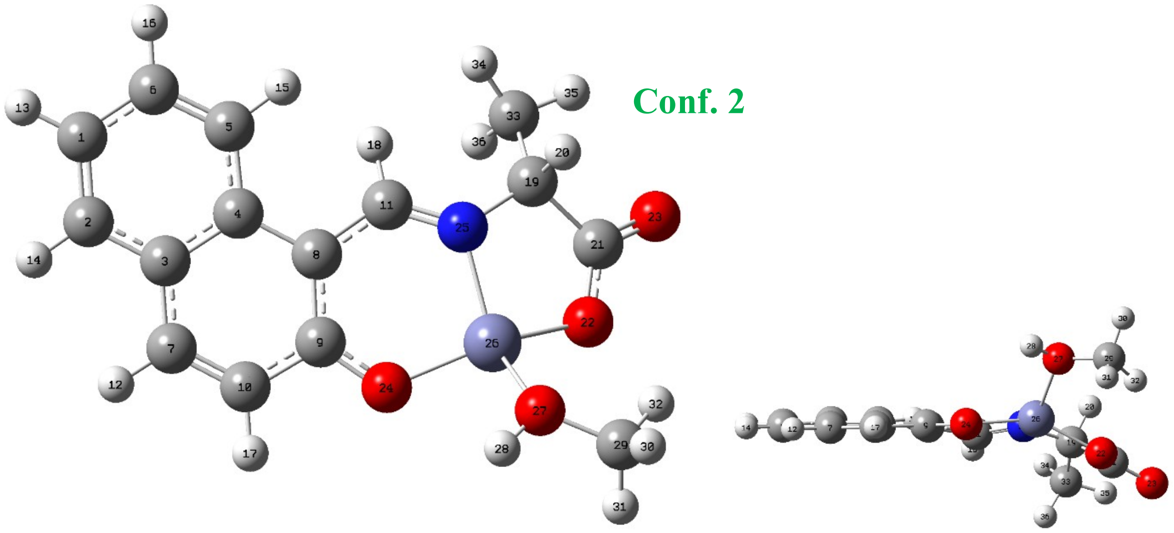

E = **-2715.254615 AU** (*G* = -2715.304614 AU)

Figure S1. Labeling of the atoms in the two main conformers of ZnAHN. Two views of the molecular structure are shown with the total energy +ZPE (*E*) and the Gibbs energy (*G*), with the most table one shown in bold.

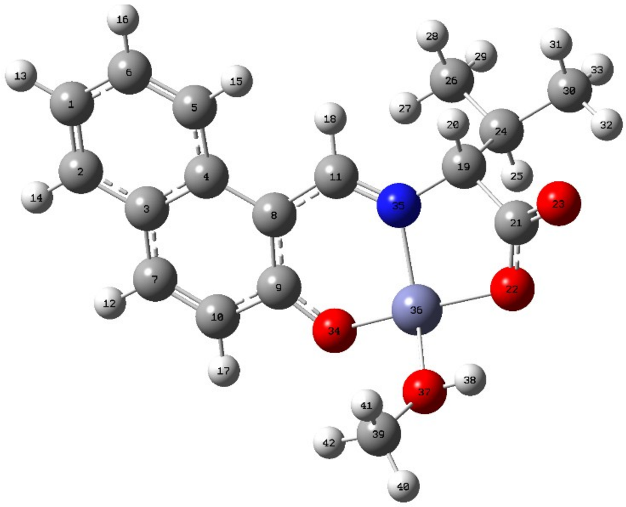

Conf. 1

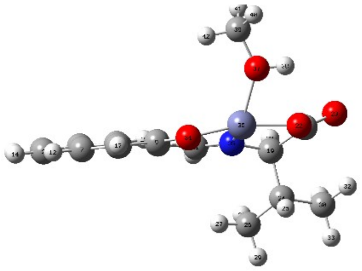

E = -2793.824606 AU (*G* = -2793.879027 AU)

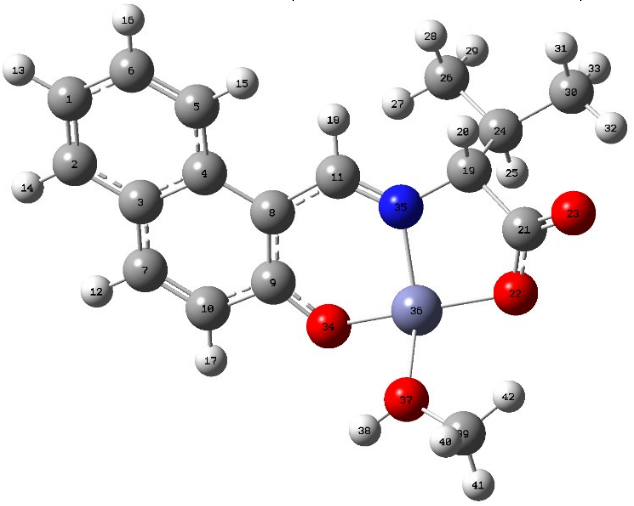

Conf. 2

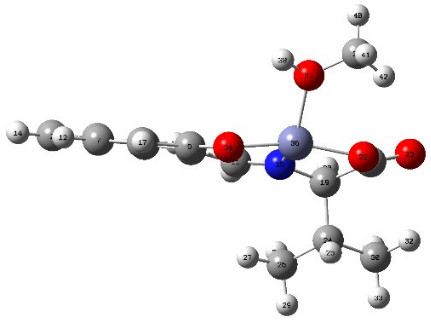

E = -2793.824448 AU (*G* = -2793.878265 AU)

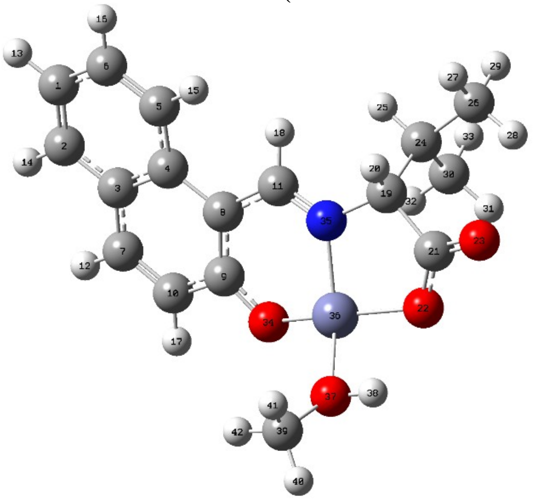

Conf. 3

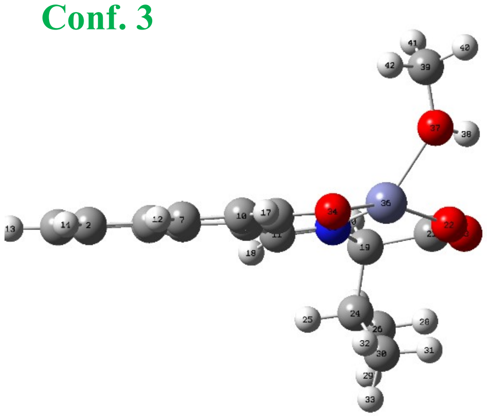

E = **-2793.828081 AU** (*G* = **-2793.882686 AU**)

Figure S2. Labeling of the atoms in the three main conformers of ZnVHN. Two views of the molecular structure are shown with the total energy +ZPE (*E*) and the Gibbs energy (*G*), with the most stable one shown in bold.

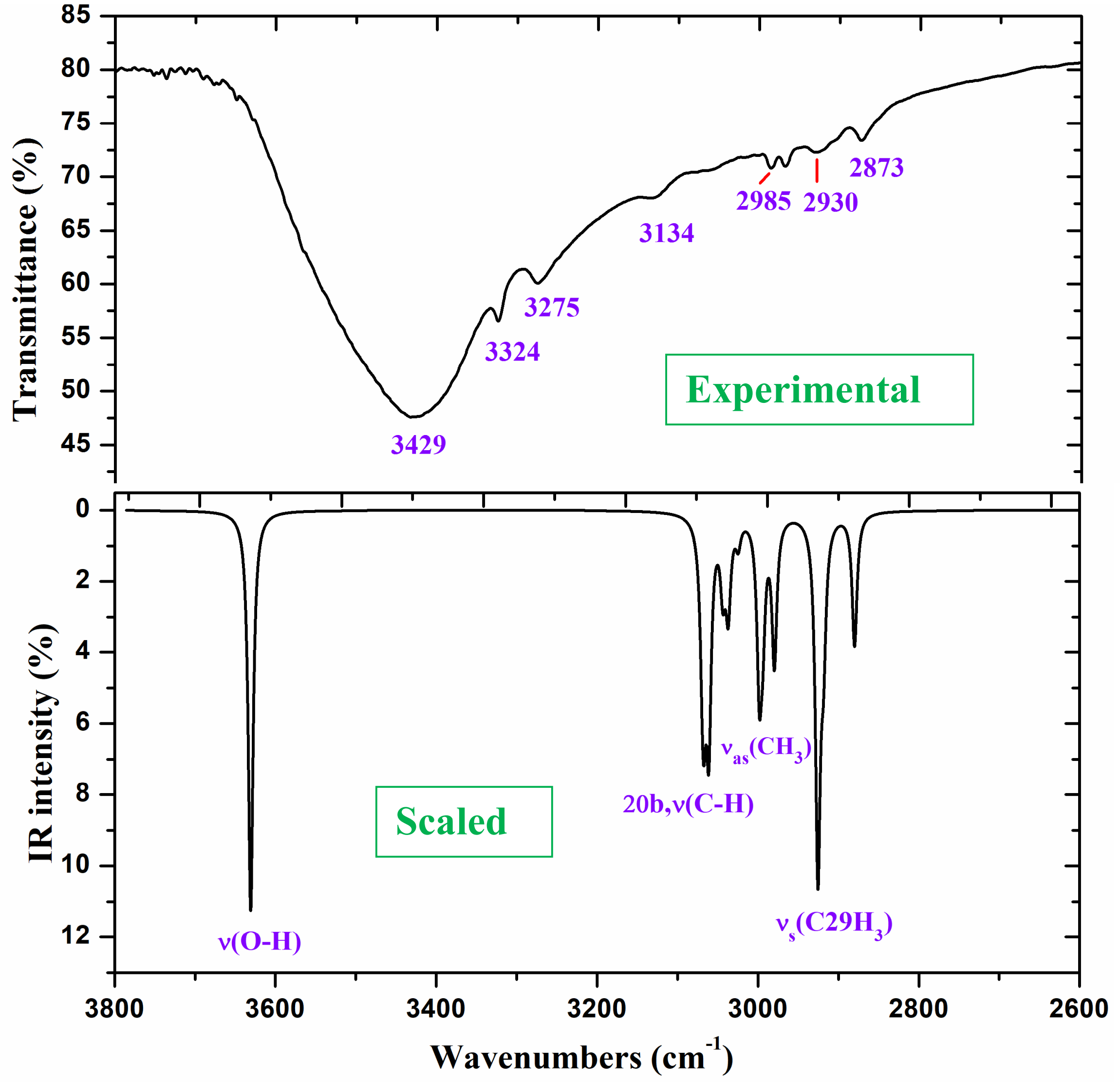

Figure. S3 Experimental IR spectrum in KBr and scaled spectrum at the B3LYP/6-31G** level of ZnAHN molecule in the 3900–2000 cm^{-1} range.

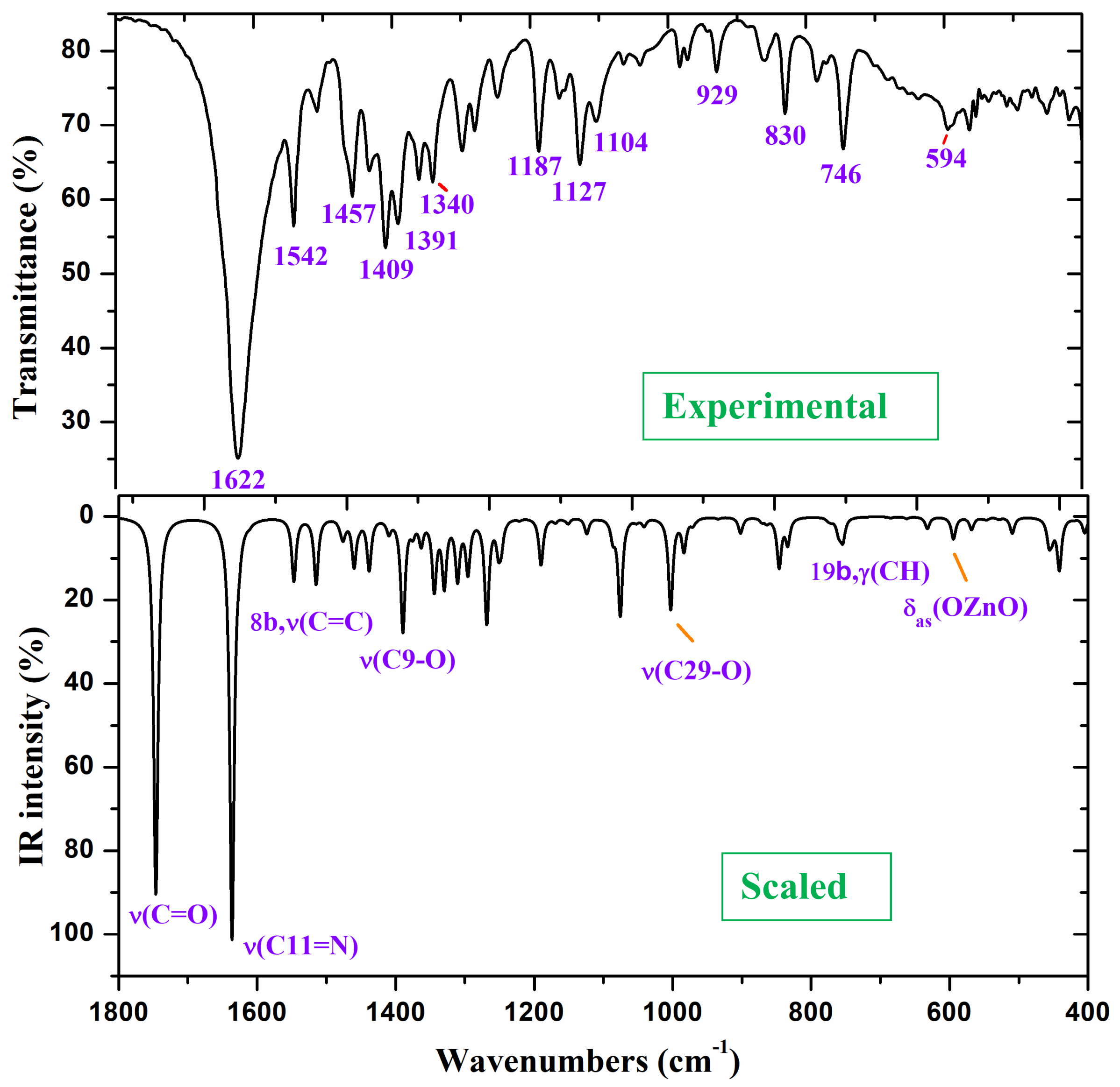

Figure S4. Experimental IR spectrum in KBr and scaled spectrum at the B3LYP/6-31G** level of ZnAHN in the 2000–400 cm^{-1} range.

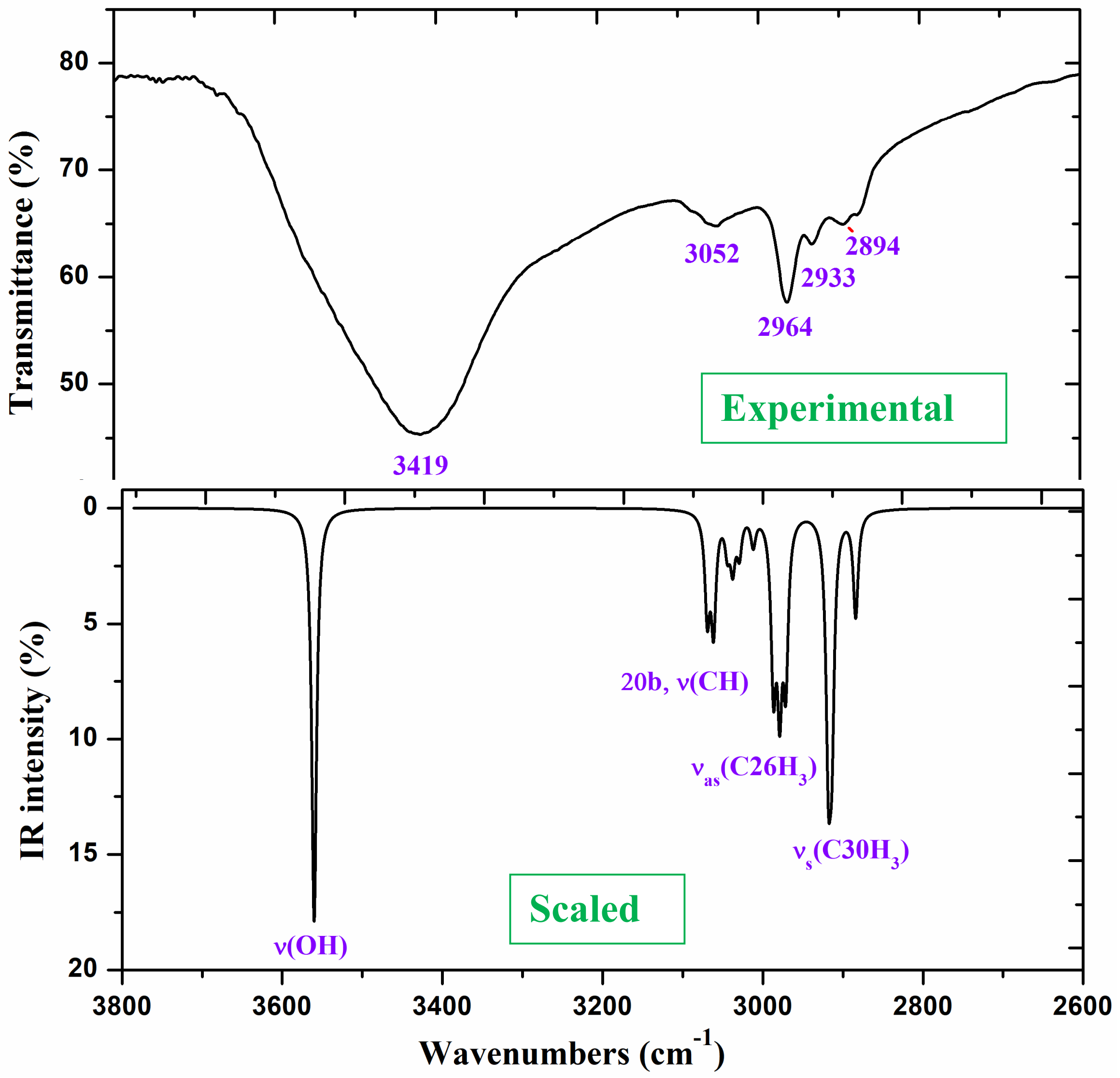

Figure S5. Experimental IR spectrum in KBr and scaled spectrum at the B3LYP/6-31G** level of ZnVHN in the 3900–2000 cm^{-1} range.

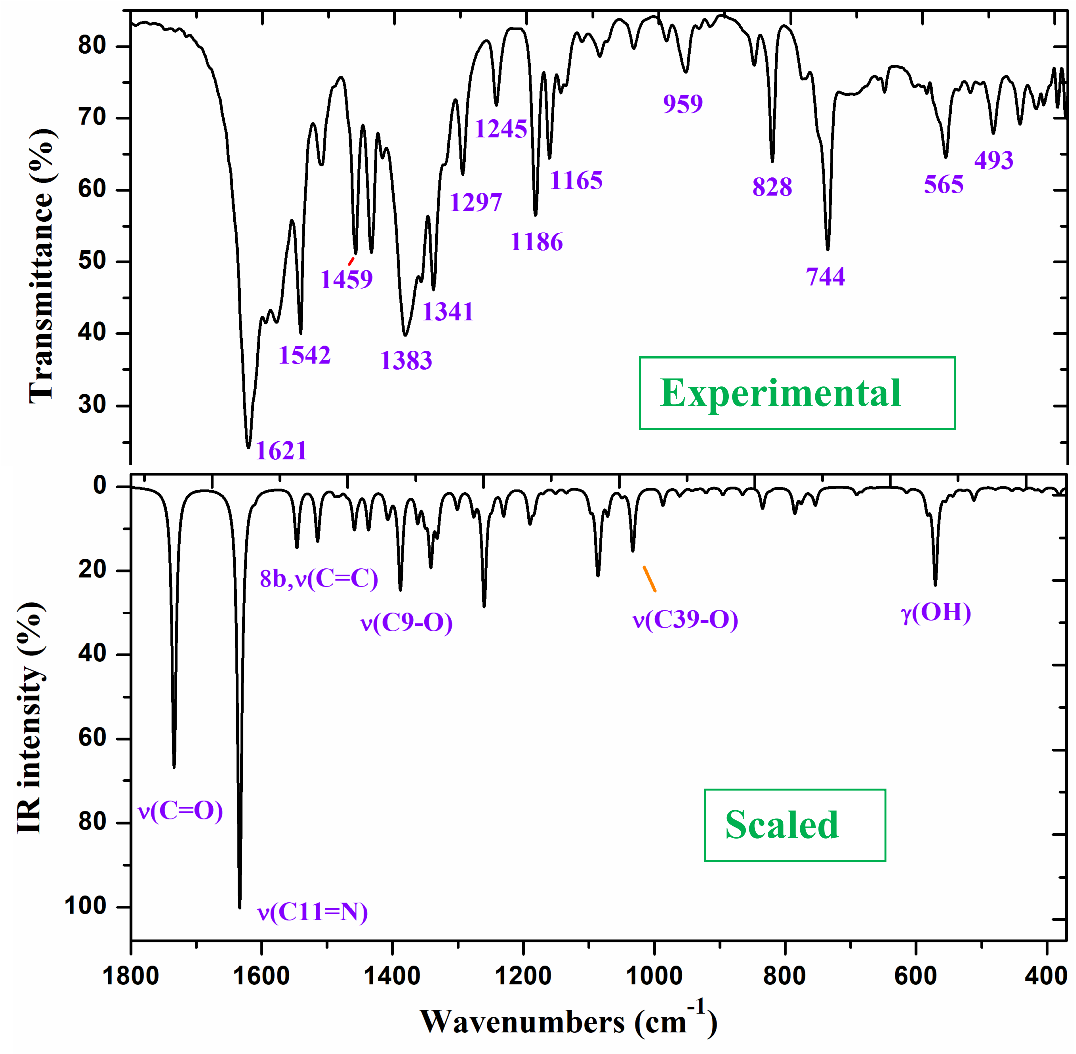

Figure S6. Experimental IR spectrum in KBr and scaled spectrum at the B3LYP/6-31G** level of ZnVHN n the 2000–380 cm^{-1} range.
